# International Medical Congress

**Published:** 1887-09

**Authors:** 


### INTERNATIONAL MEDICAL CONGRESS.

The Congress will assemble at Washington City on Wednesday, the 5th of the present month (September). There has been for the last two or three months evidences of increasing harmony and growing interest in the Congress throughout the whole country. The sections are well organized, and we may expect many able papers and a vast amount of interesting work. The local arrangements at Washington are good and a most interesting programme has been arranged both in regard to the work of the sections and to outside matters, including an excursion to Niagara and other interesting diversions.

This great body of representative men in the Profession from all parts of the world will assemble in Washington City on the 5th instant. We have read with interest a list of the many important papers that have already been announced.

The subjects treated are varied and exceedingly interesting. The Association, composed of advanced thinkers from every portion of the world, may be expected to be characterized by a dignity of action and an ability in the contributions that will far surpass the ordinary Societies and State Associations. The Book of Transactions will, therefore, be a very valuable and desirable work, illustrating the advances in the profession to the present day, and imparting to future investigations an impetus which will largely accelerate the progress of medical science in all departments.

We regard the Medical Congress as a grand and exceedingly important body in which the profession will be benefitted the world over, and which may well excite the national pride and enthuse the patriotism, not only of the profession, but of the American people at large.

